# Quinine in Croup

**Published:** 1871-01

**Authors:** H. N. Eastman

**Affiliations:** Professor of Practical Medicine in Geneva Medical College, Geneva, N. Y.


ART. II.—*Quinine in Croup.* By H. N. EASTMAN, M. D., Professor of Practical Medicine in Geneva Medical College, Geneva, N. Y.

More than twenty years ago I abandoned the legitimate mode of treating croup, as taught in the schools and authorized in the standard works, not because I regarded such treatment as absolutely erroneous or of no avail, but because I became convinced by reflection, and subsequently by observation, that there was altogether a milder, safer and better plan than was anywhere inculcated or practiced, so far as my reading and observation had extended.

Previous to this period, I had treated the disease under consideration according to the received directions of the profession; which enjoin in its early stage, the use of emetics of ipecac or antimony alone, or more frequently both combined, followed by mercurial purges, hot pediluvia or the general warm bath, topical applications, as rubefacients or hot fomentations to the throat, the various expectorants and ultimately, stimulating emetics and diffusible stimulants generally. This course I pursued for several years with variable success; generally, however, with favorable results when called in the early stage of the disease. In the meantime I lost my own eldest child—and then only daughter—after a little less than a week's illness of this fearful malady, at the age of some three and a half years.

This case began moderately and progressed gradually and insidiously, notwithstanding an early, efficient and persistent course of treatment, according to the plan above delineated, the paroxysm commencing in the night, at first, not alarmingly severe, followed next morning by nearly or quite a complete intermission of dyspnœa and the peculiar metallic cough. The case proceeded with considerable regularity in this way, evincing no positively alarming symptoms, each paroxysm however, becoming rather more severe followed by a less perfect intermission, till this last was superseded rather by a remission than an entire absence of the morbid state; this in time gave place, ultimately, to a continued or uninterrupted dyspnœa, croupy cough and general febrile action, with however, occasional though less regular and more severe exacerbations, in one of which she finally succumbed, on the fifth or sixth day after the attack.

Some eight years after this sad event, another daughter of about the same age was attacked in precisely the same way. The case progressed as nearly like that above described as might be, and that, too, under the same efficient treatment. Painfully watching the progress of the case towards a fatal result, in spite of all the active remedial measures employed, I at length lost all confidence in the plan pursued, fully convinced that the same fatal termination must, ere long, occur that carried off our first born child, unless some different and more successful plan could be put in requisition than had hitherto been adopted or recommended by the authorities. In this dilemma—reflecting on the early paroxysmal, if not positively intermitting character of the disease, as it appeared more markedly in our child, and which characterizes the ordinary forms of croup in the early stage, and bearing in mind the efficacy of quinine in subduing every form of periodical disease—I resolved at once to test its efficiency in what I regarded an almost desperate case, feeling assured that no serious deleterious effect could follow its administration, even should the remedy fail of success. Determining, if possible, to make a decisive impression on the disease, I weighed out ten grains of quinine and dividing it into two equal parts, gave one about two hours before the anticipated exacerbation, and the remaining one in an hour from the administration of the former dose. At the end of the second hours instead of the appearance of the anticipated approaching paroxysm, the child broke out in a profuse cool perspiration, was sleeping sweetly, and remained thus perfectly free from any dyspnœa or croupy cough through the night, the last quinine having been given at 7 o'clock the previous evening. In the morning the child awoke, quite free from any appearance of disease, and remained in this state, though kept in bed, through the ensuing day. At the approach of evening, lest a return of the symptoms should occur, I repeated the same course I pursued the night before. The same result followed as on the preceding night, and from this time the child convalesced rapidly without any further medication. This case, as might be supposed, made a strong impression on my mind, and I resolved to try the same remedy in the next case that might present itself. As croup was, to some extent, endemic in the region where I then resided, I did not wait long for another opportunity to test again the efficacy of the quinine treatment. Succcess was quite as well marked in the second as in the first case; and from that time forward, up to the present, I have invariably treated croup after the same plan, with as uniform success as I have met with in treating intermitting fever or any other periodical disease with this anti-periodic; indeed, I do not now recollect a single instance of croup, when I was called in any season, that is, while there remained any considerable intermissions or even remissions in the earlier part of the twenty-four hours, which nearly always occur in every form of the disease during the early stage, where I failed to subdue the malady and restore the patient at once to ordinary health. I have now just as much confidence in the power of quinine to arrest croup as I have in its efficacy in subduing any ordinary intermitting or periodical disease; and when called to prescribe for a child of any age that has suffered from attacks of croup for a day or two, as I have been this morning, I prescribe from six to ten grains of quinine to be taken in two or more doses before the hour in which the last paroxysm has set in, and with rare exceptions this is the last I hear of the case unless, on subsequently inquiring, I learn that no further return of the difficulty occurred. As to the quantity specified above, I do not hesitate to give seven or eight grains to a child six months old in any periodical disease, within a few hours. With less than this quantity I have seldom witnessed any satisfactory effect. For a child one year or more I much prefer ten grains, as this amount, given either in several divided doses during the whole period of repose or, what I think quite as well, in two or three parts at short intervals, so that the whole amount be administered at least one hour or two before the expected paroxysm or exacerbation, seldom if ever fails to entirely put a stop to the disease. I have in no instance, after the most careful observation, perceived any unpleasant effects follow the administration of quinine in such quantities to children of the ages above specified. Nor is there ordinarily any great difficulty in giving or retaining this medicine in such cases. I find altogether the most convenient way to administer the article is to rub the proposed quantity thoroughly with a convenient amount of dry sugar, to which add a given number of spoonsful of *cold tea*, according to the number of doses you propose to make of it. Of this, thoroughly stirred at the time, a spoonful may be given at the several periods decided upon, so that the whole amount shall be taken within the required time. In this way the article may be administered with little opposition on the part of the patient, and very little liability of its being rejected, provided always that you have not rendered the stomach irritable by the previous exhibition of an emetic; and for this reason I avoid, if possible, the giving of nauseating draughts or potions previous to or during the paroxysm of dyspnœa. I much prefer the warm bath, free opiates, and if the excitement be intense, a full mercurial cathartic with some simple diaphoretic, so as to preserve in this way the integrity of the stomach, for what I consider much the more important remedy, one that is essentially curative rather than palliative. If, however, the case seem imminently dangerous during the paroxysmal stage, I do not hesitate to give a powerful emetic, but one not likely to be followed by lasting nausea, and, in my experience, no article in such cases acts as well as Turpeth mineral. It is quick and efficient; Promptly relieves the laryngeal spasms: produces copious secretion of mucus from the part, powerfully equalizes the circulation, and thus obviates the vascular engorgement of the larynx and finally leaves after a short time no considerable nausea, so that within a few hours at the farthest, the quinine will be tolerated with impunity if given with the precautions above specified. Administered in grain doses, and repeated, if need be, every fifteen minutes till free vomiting ensue, it prostrates very little; never, in my hands, purges nor salivates, but leaves the stomach in a short time in a condition to bear the quinine without any difficulty. This can scarcely be said of emetic tartar or even ipecac. But in ordinary cases no emetic is required, as milder means will suffice to facilitate the passing off of the urgent symptoms, while the quinine, administered as above, effectually prevents the recurrence of the disease.

As in intermitting fever, I consider the object of treatment to consist less in subduing the existing paroxysm, which in all ordinary cases ere long passes off of itself safely, than in effectually preventing a return of the morbid condition; so in croup, I am more solicitous to anticipate the succeeding paroxysm than to overcome the present one, which seldom proves fatal or even dangerous. Such a course of treatment, I am entirely persuaded, will, in nearly every case of croup, supersede the necessity of resorting to violent emetics, revulsives, mercurilization, tracheotomy, "and all that sort of thing," as recommended by "the books," and, to say the least, by a large proportion of the teachers of practical medicine; not that the remedies authoritatively prescribed are valueless or inefficient in equalizing the circulation and thus restoring normal action, the principal on which all these means act, but that there is a milder, safer, surer and therefore better way to accomplish the desired end. I believe there is a radical error made by the authorities in dividing croup into two distinct forms—spasmodic and inflammatory or pseudo-membranous; one that too often leads to fatal mistakes in its treatment—the former being regarded as a comparatively not dangerous disease, while the latter is considered a most frightful malady, and one, too, that is scarcely amenable to any treatment. The effect of such views is to render him who adopts them unconcerned and indifferent in prescribing for what he judges to be spasmodic or catarrhal croup on the one hand, and on the other, quite as inert and all but despairing in attempting to combat the more serious form, or rather the alarming stage, or, as he regards it, true inflammatory or membranous croup. In the one case, the physician loses a most valuable opportunity, if not the only chance of subduing the morbid action "*in limine*," and thus preventing what is always liable to follow—a true inflammatory stage—and ultimately finds himself vainly striving to overcome a state that can scarcely yield to the more judicious and heroic treatment, one that an early and wise course might have effectually obviated. The truth is, both forms of croup, so-called, are but different stages of the same disease, viz.:— a vascular engorgement, and, it may be, subsequent effusion taking place in the larynx, extending in some instances into the trachea, and, rarely, into the bronchial tubes—in other words, a laryngo-tracheitis. All the urgent symptoms are referrable to the larynx, the origin of the morbid action, in consequence of the exceedingly narrow aperture of the rima glottidis and consequent impediment to the ingress of air, in consequence both of a morbid thickening of the mucous membrane of the part, or subsequent effusion, and still more, it may be, from spasmodic action of the laryngeal muscles, always present to a greater or less extent, as an *effect* of the vascular disturbance rather than the cause.

In the language of Professor Peaseley, of New York, in his admirable note under the lecture on croup in "Watson's Practice," "All croup is inflammatory," and he might, with equal propriety have added, all croup is spasmodic. The first appreciable link in the chain of morbid action is congestion of the vessels of the larynx, just as congestion is the starting point in all inflammatory states, a simply engorged condition of the capillaries of the part, from some cause not necessary now to be accounted for, a state attended at first with a temporary arrest of the normal secretions. Hence the preternatural dry cough in croup, characteristic of the disease in its early stage, subsequently with a thickening of the laryngeal mucous membrane, producing the dyspnœa that soon follows; and finally, if the case goes on or passes into the inflammatory stage with an effusion of liquor sanguinis on the mucous surface, which either degenerates into pus, as in ordinary croup, and which readily escapes by expectoration, deglutition or vomiting in case of infants; or in extreme cases coagulates on the free surface, forming a false membrane or cast of the part as it thickens by accretion, which effectually, in the end, fills the narrow chinck, obstructing the passage of air to and from the lungs, rapidly producing death by apnœa, unless removed by violent coughing, or the action of a prompt stimulating emetic, as sometimes happens, but which too often returns, repeatedly it may be, till finally the helpless sufferer, worn out and exhausted by ineffectual efforts of respiration, and poisoned by imperfectly decarbonized blood, sinks and dies.

Throughout all the several stages the laryngeal muscles are, at times, thrown into more or less violent spasms, greatly aggravating, for the time, the dyspnœa and agony of the patient, and not unfrequently proving suddenly fatal by the protracted apnœa occasioned thereby; such spasms are but the effect of a preëxisting abnormal state of the vascular tissue of the part, just as spasmodic action of the circular fibres of the minute bronchial tubes at times complicate bronchitis, producing dyspnœa or asthmatic paroxysm of breathing which every one has witnessed, but which no one would regard as the original malady.

There is a purely spasmodic disease of the larynx, termed laryngismus stridulus, but this usually comes on suddenly; there may be one or more paroxysms which abate as suddenly, leaving the patient in the meantime free from dyspnœa, croupy cough and fever. Rarely, this disease continues to recur in distinct paroxysms, more or less frequent, for one or two weeks even, in spite of all remedial measures, depending, it may be, on some lesion of a distant nerve or nerve centre, till finally the little patient succumbs from sheer exhaustion. I have witnessed one such case, but this, as every intelligent physician knows, differs from croup proper in everything, save the mere accident of laryngeal spasms, which, in the latter, occurs as one of the symptoms merely, an effect produced by a preexisting congestion or inflammatory condition of the larynx.

I contend that there is no essential difference between croup in children and laryngitis of maturer years, though each is marked by peculiarities incident to this circumstance of age. In croup or puerile laryngitis the effusion, whether the simple serum of congestion or the liquor sanguinis of the inflammatory stage, always I think, is thrown out upon the free surface or mucous membrane of the larynx. On the other hand, in adult laryngitis the same effusion, whether watery merely, or coagulable lymph, is poured out in the sub-mucous tissue, causing œdema glottidis, which almost necessarily produces rapid dissolution unless speedily obviated by surgical interference. Why this is so I cannot tell, unless it results from the peculiarity of structure existing at the different ages. Again, in croup, or laryngitis of children, there is a strong propensity for the phlogistic condition to extend downward into the trachea, and even in some extraordinary cases, into the small bronchial tubes, and for this reason tracheotomy very generally perhaps, proves useless from the fact that the proposed relief fails to reach below the extent of the diseased action; whereas in laryngitis of more advanced age, the inflammation seldom if ever extends below the original seat of the disease, consequently this operation, if resorted to seasonably and performed skillfully, very generally effects a cure by affording an opportunity for free respiration for any requisite period, while the primary disease has time to subside spontaneously or by the aid of appropriate remedies.

In many instances of croup the congestion, arising in almost all cases from cold, subsides spontaneously in a few hours, or yields to the administration of the mildest remedies, not again to return, without a repetition of the exciting cause. In most cases, however, the paroxysm reappears about twenty or twenty-four hours after the first attack, with an interval, it may be, of complete intermission of all the symptoms—that is, when the case is left to itself or is ineffectually treated. These paroxysms, if they are allowed to succeed each other, as seems to be their general tendency, become more severe and protracted, while the intervening intermissions in time are shorter and less perfect, till the case becomes one of mere exacerbation and remission, or even continuous; in other words, the periodical congestions of the larynx pass into positive inflammation, or that state of continued vascular engorgement characterized by effusion of liquor sanguinis, depositing purulent material or false membrane, as the case may be, on the free surface or in the cellular tissue beneath the mucous membrane of the larynx, according as the subject may be a child or an adult. We now have the inflammatory stage, or what is called catarrhal or membranous croup, with all the train of alarming symptoms that are diagnostic of this terrible stage—a stage that but too often proves fatal under any treatment. While this is the general course of croup, whether it subsides favorably or goes on to a fatal termination, there is another form that rarely occurs, but which, though apparently to an unskilled observer much less formidable in its incipient stage, is nevertheless, far more alarming and one which, in too many instances, goes on insidiously but uninterruptedly to a fatal termination, in spite of the most prompt and judicious treatment.

In other cases, the stage of congestion or simple vascular engorgement is so slight and transient that it is generally overlooked or passed by before the physician is called in, and the true inflammatory condition, or that of continued capillary distention with effusion of coagulable material is already well established, a state of things, as already indicated, well nigh hopeless; for in croup more than in almost any other local ailment, the time to effect a cure is emphatically during the congestion or forming stage, before liquor sanguinis is poured out and further structural changes occur, a stage which in all ordinary cases intermits, or at least remits, repeatedly before the phlogistic stage proper sets in; and it is during these intermissions or remissions that the judcious employment of appropriate remedies is most efficacious and reliable. If this stage be suffered to pass unheeded, or be ineffectually treated, all after measures will be found, in a majority of cases, nugatory. Hence the importance of correct views of the pathology of croup, and hence my objection to the ordinary plan of dividing it into two distinct forms, rather than simply two stages of the same disease. This erroneous notion of two distinct forms of croup naturally leads those who adopt it to a neglect, or an insufficient treatment, of the so-called spasmodic croup which, I think I have shown to be, only the forming stage of a true inflammatory condition of the part, or a laryngitis, and the only one in which we can entertain a well-grounded hope of an effectual cure, a hope which rarely fails to be realized. If, unfortunately, we are called too late to take advantage of the periodical state of the disease, other means must needs be resorted to, to arrest, or remove, if possible, the deposition on the free surface, or in the sub-mucous tissue, by topical applications, possibly mercurialization, prompt stimulating emetics, inhalations of medicated or simple vapor, and finally, it may be, tracheotomy. But even in cases where the stage of deposition has already set in, I would place more reliance on a continued exhibition of free quantities of quinine, given as an antiphlogistic, than on any other measure. Here large quantities only are available. I would not administer less than from fifteen to thirty grains in twenty-four hours, to a child a year old or over. In these desperate cases it requires double the amount of quinine to subdue an existing inflammation and arrest the further effusion of lymph that is needed to prevent the deposition of coagulable matter during the intermissions or remissions that occur in the congestive stage, on the principle that "an ounce of prevention is worth a pound of cure." Nor is there anything to be apprehended from this heroic use of the article in violent cases of croup in young children in the latter stage. I have resorted to it in some few cases with complete success, and never with any alarming or untoward symptoms. Indeed there seems to be almost a complete tolerance of this wonderful remedy in croup or puerile laryngitis.

Dr. McFarlane, now of New York, reported a case, some twenty years ago, in *The New York Journal of Medicine*, in which was administered, through mistake, some thirty-five grains of quinine in twenty-four hours, to a child five years old, in croup, rather as a support or stimulant in the sinking stage, it would seem, than as an antiphlogistic, and that too without any injurious effects, but, as it proved, with complete success. But I repeat, the true mode of successfully treating croup in children, and also laryngitis, as it is termed, in mature age, is to administer moderate but fresh quantities of quinine during the intervals of the congestion or forming stage, so as to prevent a recurrence of the paroxysm or exacerbation, just as we give the same article in the intermission or remission of periodical fever or neuralgia, to forestall the succeeding paroxysm; and I affirm, with a confidence resulting from twenty years' uniform trial of this course, that the medicine is quite as reliable in the one case as the other. Perhaps other anti-periodicals, as arsenic, would produce the same effect. I have not tried them, having been perfectly satisfied with the effect of quinine, a perfectly innoxious substance when administered with ordinary prudence. Possibly a a less quantity of quinine would suffice to produce the desired effect on children of the ages above specified, but after repeated trials I have failed to accomplish the object in view with a less quantity than that above stated. I do not know that it makes any essential difference whether the amount be given in divided doses through the whole interval, from the subsidence of a paroxysm to the approach of the succeeding one, or exhibited in two or three large doses, at shorter intervals, so as to secure the full effect of the last, before the inception of the anticipated return of the congestion. I have pursued both courses with equal success. I have not only pursued this plan of treatment myself, with uniform success for the last twenty yeaas, but many of my professional friends have been induced, by my suggestions, to make trial of the same means, and, so far as I am informed, all who have tried the course have been convinced of its superior excellence to that of the common treatment, as taught in the books and in most of our schools, and practiced by the great body of the profession. I have publicly inculcated these views in my lectures for the last several years, during which time I have received innumerable letters from graduates, after having repeatedly tested the truth of the doctrine I had taught, all of which gave expression of their fullest confidence in the success of their trials.

Recently I have been not a little surprised and gratified in learning thatother practitioners, of high standing and large experience, have been successfully treating croup after the plan recommended above, for some time past, and that, too, without any concert of action or any knowledge of each others course, another instance in which men from careful observation and earnest thought arrive at the same conclusion without any previous knowledge of each others experience or course of reasoning. I do not repudiate the teachings nor undervalue the success of our predecessors, or the great body of practitioners who still adhere to the old doctrices and practice, in their treatment of croup. The general anti-phlogistic treatment, so-called, as bleeding, emetics, mercurial cathartics expectorants, revulsions, etc., tends more or less powerfully to equalize the circulation and thus overcome congestion of the part, and restore the healthy action; and, I doubt not, this course was attended with general satisfactory results, more so probably in former times than would be at the present, when patients illy bear depletion. Indeed, I know this to be the case, from my own personal experience, in the early part of my practice some thirty years ago. I only urge that while such a course often succeeded, its ultimate tendency is to reduce and lower the vital forces rather than to repair or exalt them, and on that account, that it is more or less objectionable, according to the circumstances of the case; that there is a shorter, surer, safer and therefore better way, one that while it is more efficient in restoring the equilibrium of the circulation and overcoming the local disease, is restorative in its operation, entirely harmless in its tendencies, and for these reasons, should supersede all less reliable means.

